# Roles of Oxidative Stress in Synaptic Dysfunction and Neuronal Cell Death in Alzheimer’s Disease

**DOI:** 10.3390/antiox12081628

**Published:** 2023-08-17

**Authors:** Germán Plascencia-Villa, George Perry

**Affiliations:** Department of Neuroscience, Developmental and Regenerative Biology, The University of Texas at San Antonio (UTSA), San Antonio, TX 78249, USA; george.perry@utsa.edu

**Keywords:** Alzheimer’s disease, oxidative stress, oxidative damage, free radicals, neurodegeneration, oxidants, antioxidants, neurons

## Abstract

Alzheimer’s disease (AD) is a brain disorder that progressively undermines memory and thinking skills by affecting the hippocampus and entorhinal cortex. The main histopathological hallmarks of AD are the presence of abnormal protein aggregates (Aβ and tau), synaptic dysfunction, aberrant proteostasis, cytoskeletal abnormalities, altered energy homeostasis, DNA and RNA defects, inflammation, and neuronal cell death. However, oxidative stress or oxidative damage is also evident and commonly overlooked or considered a consequence of the advancement of dementia symptoms. The control or onset of oxidative stress is linked to the activity of the amyloid-β peptide, which may serve as both antioxidant and pro-oxidant molecules. Furthermore, oxidative stress is correlated with oxidative damage to proteins, nucleic acids, and lipids in vulnerable cell populations, which ultimately lead to neuronal death through different molecular mechanisms. By recognizing oxidative stress as an integral feature of AD, alternative therapeutic or preventive interventions are developed and tested as potential or complementary therapies for this devastating neurodegenerative disease.

## 1. Introduction

The human brain contains around 86–100 billion neurons and 10 times more glial cells, with approximately 0.15 quadrillion (0.15 × 10^15^) synaptic connections in the neocortex [1,2]. Neurons are unable to efficiently renew themselves due to their terminally differentiated post-mitotic state, which occurs very early during development, and remain alive and functional for decades [3]. Evidence of adult hippocampal neurogenesis has remained elusive, indicating that neurogenesis could occur in healthy subjects but declined in subjects affected by neurodegeneration [4]. Consequently, brain cells need efficient molecular machinery to maintain and repair cell integrity. Aging is accompanied by mild changes in the human brain, including the collapse of the structure and function of neurons and glia cells; alterations in metabolism, biochemistry, and cell organization contribute to progressive cognitive decline [5]. Under normal conditions, these changes also include brain volume loss, dysfunction in the cholinergic system, memory decline, and alterations in gene expression and synaptic function. In contrast, in neurodegeneration, these cellular and molecular changes are more dramatic, compromising the overall integrity and function of the brain. The progressive deterioration of the brain leads to the collapse of the structure and function of neuronal circuits, causing impaired memory, cognitive decline, altered behavior, and abnormal motor function.

**Figure 1:**
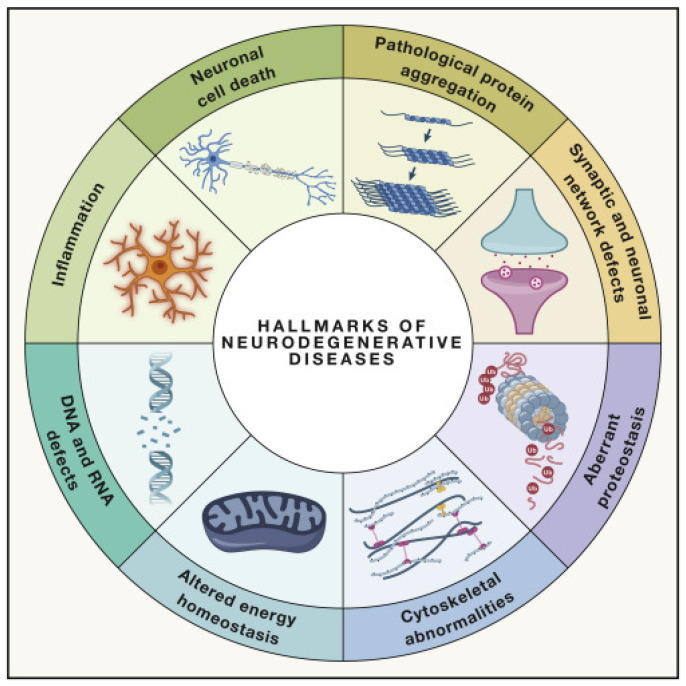
Hallmarks of neurodegenerative diseases. The main histopathological hallmarks identified in Alzheimer’s disease and related dementia are (1) pathological protein aggregation (Aβ and tau), (2) synaptic and neuronal network dysfunction, (3) aberrant proteostasis, (4) cytoskeletal abnormalities, (5) altered energy homeostasis, (6) DNA and RNA defects, (7) neuroinflammation, and (8) neuronal cell death. Reproduced from [6].

Alzheimer’s disease (AD) is a chronic and progressive neurogenerative disorder affecting the brain cortex and hippocampus, causing synaptic alteration, neuronal failure, memory and cognitive impairment, and irreversible loss of neurons. Even through decades of research, the etiology of AD is not fully understood, but aging is the main contributor in combination with genetic, environmental, and lifestyle factors. This neurological disorder is accompanied by several changes in the brain metabolism and anatomy, especially in the affected brain areas and subcellular populations. Neurodegenerative disorders, such as Alzheimer’s disease, are characterized by distinguishable histopathological hallmarks (Figure 1), including (1) pathological protein aggregation (e.g., Aβ and tau), (2) synaptic and neuronal network dysfunction, (3) aberrant proteostasis, (4) cytoskeletal abnormalities, (5) altered energy homeostasis, (6) DNA and RNA defects, (7) neuroinflammation, and (8) neuronal cell death [6]. Another hallmark commonly found in AD and related dementia is *oxidative stress* or *oxidative damage*, but it is frequently overlooked or considered a consequence of one or more of the main AD histopathological hallmarks. Remarkably, oxidative stress is directly or indirectly linked to each one of the AD common features, and the signs of oxidative stress are present from the earliest stages of the neurological condition; however, the etiology is still poorly understood.

The therapeutic approaches for AD have focused on maintaining or stabilizing brain function by regulating neurotransmitters (cholinesterase inhibitors and N-methyl D-aspartate antagonist), removal of Aβ aggregates (therapeutic monoclonal antibodies), and management of agitation symptoms (brexpiprazole) [7]. Alternative therapeutics for AD are focused on targeting oxidative stress to reestablish or control the antioxidant systems in the brain, removal of free radicals and reactive oxygen species, or to promote the renewal of oxidized molecules.

Here, we present an overview of the roles of oxidative stress in AD and related dementia, including the effects of oxidative damage to susceptible neurons and brain areas affected by AD, particularly in triggering programmed cell death responses. Moreover, we discuss alterative therapeutic approaches or supplements designed to control oxidative stress in the brain that could be combined with the already FDA-approved AD drugs.

## 2. Effects of Amyloid-β Peptide on Neuronal Oxidative Stress

**Figure 2:**
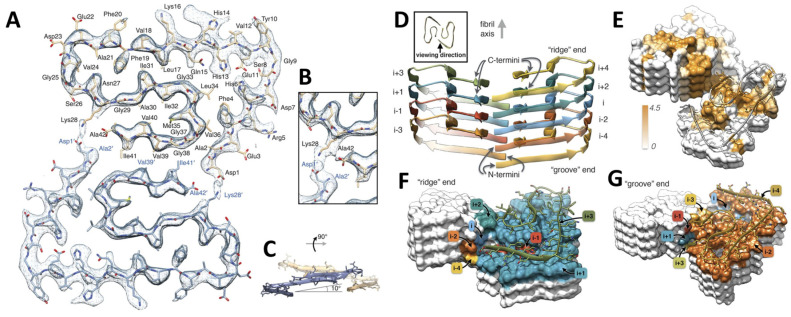
Structure of amyloid-β (1–42) peptide in fibrillar aggregates. (**A**) Atomic-resolution structure of Aβ_1–42_ superimposed with cryo-electron microscopy density map. (**B**) Inset shows a detailed view of the interactions between the -*N* and -*C* terminus (Asp1 and Ala42) with the side chains of Lys28. (**C**) Side view of the opposing subunits showing the relative orientation of the nonplanar subunits; the cross-β sheets are tilted by 10° with respect to the plane perpendicular to the fibril axis. (**D**) Side view of Aβ_1–42_ fibrils showing the staggered arrangement of the nonplanar subunits. (**E**) Surface representation of Aβ_1–42_ fibrils according to hydrophobicity (Kyte–Doolittle scale), orange–brown areas (hydrophobic, 4.5) to white (neutral, 0.0). (**F**) Surface view of the “ridge” fibril ends. (**G**) Surface views of the “groove” fibril ends. Reproduced with permission from [9].

The main histopathological hallmark of AD is the presence of abnormal aggregates of Aβ peptide in amyloid plaque cores (APCs). The overexpression and accumulation of APC in the affected areas of the brain (prefrontal cortex, hippocampus) have been linked to neurotoxicity, inflammation, synaptic dysfunction, mitochondrial dysfunction, and progressive neuronal death. Aβ actively contributes to the progression of AD through complex antioxidant or pro-oxidant mechanisms, directly impacting oxidative stress responses [8]. Aβ is a short peptide of 40–42 amino acids that forms twisted protofilaments (7 nm in diameter) with a parallel cross-β structure and contains hydrophobic clusters that stabilize the conformation into Aβ fibrils (Figure 2) [9]. The Aβ fibrils aggregate into APC with a round radiating structure with protruding spikes. 

### 2.1. Amyloid-β Peptide Pro-Oxidant Activity

The oxidative properties of Aβ have been observed in vitro and in vivo, promoting oxidative damage to neurons. The toxicity of Aβ results from free radical damage, particularly by an increase in H_2_O_2_ and lipid peroxides in cultured neurons [10]. Moreover, Aβ induces the activity of NF-κB, which regulates oxidative stress responses. However, the direct production of H_2_O_2_ by Aβ is under scrutiny because the accumulation of peroxides is possibly related to compromised mitochondrial activity, reduced intracellular ATP concentration, and impairment of energy homeostasis [11]. The fragment Aβ_25–35_ increases the levels of free and protein-bound 4-hydroxynonenal (HNE), a neurotoxic lipid in cultured hippocampal neurons [12]. HNE mediates Aβ_25–35_ pro-oxidant activity and promotes oxidative damage to neuronal membranes and proteins. In oligodendrocytes, Aβ_40_ and Aβ_25–35_ promote DNA fragmentation, mitochondrial dysfunction, and cytoskeletal damage, including the activation of redox-sensitive transcription factors NF-κB and AP-1 [13]. Also, in rat hippocampal and cortical neurons, the peptides Aβ_40_, Aβ_25–35,_ and FeSO_4_ induce lipid peroxidation and impairment of glucose transport. As a consequence, neurons suffer ATP depletion and cell death, but in the presence of antioxidants (n-propyl gallate and vitamin E), these effects are significantly reduced [14]. In primary cultures of human fetal neurons, Aβ_1–42_ treatments (0.5–50 μmol/L) caused a time-independent and concentration-dependent reduction of electrophysiological responses (whole-cell outward currents) and significant neurotoxicity, particularly inducing caspase-dependent and caspase-independent apoptotic cell death [15]. Primary hippocampal cells from a rat AD model showed oxidative stress responses to Aβ_1–42_ and Aβ_1–42_ combined with Fe(II)+buthionine sulfoximine, indicating that Aβ formulations triggered prooxidant aspects of AD pathogenesis [16]. The neurotoxic effects of Aβ are enhanced by the prooxidant compounds Fe(II)+buthionine sulfoximine, indicating a synergistic effect to damage hippocampal neurons and glia cells. The effects of Aβ result in exacerbated mitochondrial oxidative stress in neurons, particularly by downregulation of neuronal mitochondrial antioxidant defense pathways, including glutaredoxin (*GLRX*), glutathione peroxidase (*GPX*), glutathione reductase (*GSR*), glutathione transferase (*GST*), superoxidase dismutase (*SOD2*), thioredoxin (*TXN2*), and thioredoxin reductase (*TXNRD*), among other genes of the antioxidant defense system [17].

Aβ assembled into fibrils is recognized as neurotoxic, particularly by overproduction of free radicals such as reactive oxygen species (ROS), protein oxidation, lipid peroxidation, and neuronal dysfunction [8], and through its capability to form calcium-permeable pores [18]. Oligomeric Aβ promotes the production of reactive oxygen species in neurons, causing impairment of NMDA receptors, and is linked to mitochondrial dysfunction [19]. Fibrillar Aβ showed neurotoxic effects on human primary neurons, inducing cell death through NADPH oxidase-mediated activation and production of superoxide radicals [20]. Remarkably, the aggregation of Aβ seems to be mediated or catalyzed by metal ions (Cu^2+^, Fe^2+/3+^, Zn^2+^, and Al^3+^) that may contribute to the oxidative activity and overproduction of free radicals and hydroxyl radicals [21,22,23,24]. In the presence of Fe^2+^ and Cu^2+^, Aβ peptide shows enhanced production of ROS [25], and in the presence of the iron chelator deferoxamine, neuronal toxicity is significantly attenuated [26].

### 2.2. Amyloid-β Peptide Antioxidant Activity

In contrast, under some circumstances, Aβ shows antioxidant properties. Aβ_40_ efficiently inhibited the auto-oxidation of lipoproteins and plasma but with no effect on LDL oxidation induced by excess free Cu(II) ions [27]. In contrast, in equimolar concentrations of Aβ_40_–Cu(II), the metal-induced oxidation of LDL was delayed due to the metal-chelating antioxidant property of amyloid peptide. Other Aβ peptides have shown antioxidant activity in the order Aβ_40_ > Aβ_42_ > Aβ_25–35_. Furthermore, the oxidative resistance levels of CSF are correlated with the amount of Aβ_40_, Aβ_42,_ and ascorbate, and inversely with the amount of fatty acids [28]. Amyloid peptide fragment (Aβ_25–35_) modulates lipid peroxidation in a dose- and time-dependent manner; the residue Met35 seems to have a central role, serving as a free radical scavenger or metal-binding site [29]. The oxidation of Met35 residue (formation of sulfoxide) affects the assembly and aggregation properties of Aβ [30]. Moreover, an increase in Aβ deposition in AD, PD, and Down syndrome correlates with a decrease in 8OHG [31]. In the brain of patients with Down syndrome, the signs of neuronal oxidative stress occur prior to the deposition of Aβ. The accumulation of oxidized nucleic acids (8OHG) and oxidized proteins (nitrotyrosine) in susceptible neurons seems to have started a decade earlier than Aβ deposition [32]. Reactive His residues of Aβ_1–42_ have shown redox activity by promoting a reduction of ascorbate oxidase activity and formation of ascorbate radicals when free Cu(II) or Fe(III) are present [33]. Aβ_1–42_ functions as an antioxidant to prevent Cu(II) catalyzed oxidative reactions and lipid peroxidation; this occurs in complexes of 2:1 molar ratio Aβ_1–42_:Cu(II) because in equimolar or excess Cu(II), an oxidant activity is observed [34]. 

Overall, the antioxidant properties of Aβ arise when it is part of lipoprotein complexes, binding or removing redox-active metal ions and preventing further oxidative reactions [8]. When Aβ is overproduced or not correctly processed (amyloidogenic pathway) under oxidative stress conditions, then it will form fibrils with neurotoxic and oxidative properties.

## 3. Oxidative Damage to Susceptible Neurons

**Figure 3:**
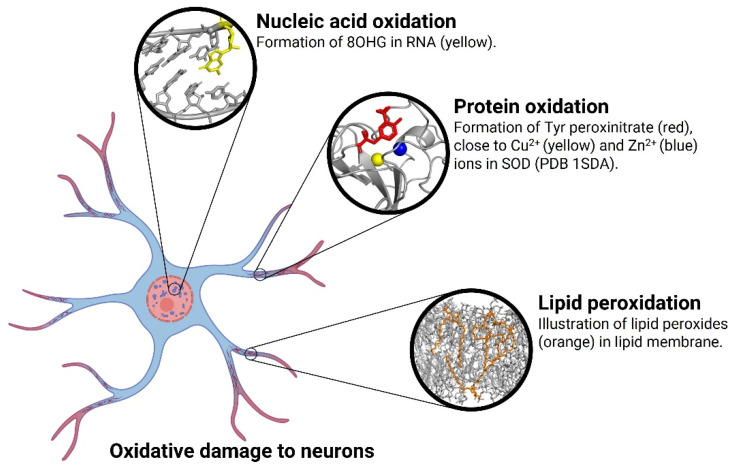
Oxidative damage to neurons in Alzheimer’s disease.

Oxidative stress refers to a cellular state with alterations in the antioxidant system, characterized by an imbalance between the production and accumulation of reactive oxygen species (ROS), reactive nitrogen species (RNS), and other highly reactive free radicals [35]. In AD, glucose metabolism is altered, especially the activities of glucose-6-phosphate dehydrogenase and 6-phosphogluconate dehydrogenase, which contribute to elevated levels of peroxides in affected neurons [36] (Figure 3). 

### 3.1. Oxidative Damage to Proteins

Oxidative damage is a key feature of Alzheimer’s disease, including alterations to enzymes in glycolysis, the tricarboxylic acid cycle, and ATP biosynthesis [35]. The signs of oxidative damage start from the mild cognitive impairment (MCI) or prodromal AD stage. Proteins from CSF of MCI patients showed an increase in carbonylated levels, especially gelsolin, vitamin D-binding protein, alpha-1 antitrypsin, alpha-1B glycoprotein, apolipoprotein E, and prostaglandin-H2 D-isomerase [37]. These proteins remain oxidized in the AD stage, indicating that the oxidative damage initiates and remains during the course of the neurodegenerative disease. Oxidized proteins are also present in the MCI hippocampus [38], especially in proteins involved in energy metabolism, synaptic plasticity, and mitogenesis/proliferation, such as α-enolase, glutamine synthetase, pyruvate kinase M2, peptidyl-prolyl cis/trans isomerase 1, hypoxia-inducible factor 1, plasminogen, MYC, tissue plasminogen activator, and BCL2L1, which may play a metabolic role in the progression of MCI to AD. Biomarkers of neuronal protein oxidation (W/S ratio of MAL-6 spin-labeled synaptosomes, phenylhydrazine-reactive protein carbonyls, glutamine synthetase activity, and creatine kinase activity) are significantly altered in brain regions affected by AD (cerebellum, inferior parietal lobule, and hippocampus). Particularly, there is an increase in protein carbonyls and a reduction in glutamine synthetase and creatine kinase activity, which are correlated with brain areas of Aβ deposits and with reactive microglia [39].

Another protein damage is due to peroxynitrate formation, which occurs by reaction of superoxide radicals with nitric oxide; Tyr residues are nitrated to peroxynitrate (Figure 3). The protein nitration is present in neurons in brain areas at stage of death, especially in the hippocampus and at a lower level in the cortex [40].

### 3.2. Nucleic Acid Oxidation

The uncontrolled production of ROS and RNS inside cells leads to the oxidation of nucleic acids (Figure 3). Guanine resides in DNA are oxidized to 8-hydroxy-deoxyguanosine (8OHdG), whereas in RNA, 8-hydroxyguanine (8OHG) is generated; both are biomarkers of oxidative damage to nucleic acids in susceptible cells. These are not the only base oxidation products, but are the most commonly found in oxidative stress conditions. 

The ratio of 8OHG levels in intact DNA to free 8OHG in the ventricular CSF of subjects with AD is significantly increased in comparison with age-matched controls [41]. The difference between AD and non-AD is dramatic, with an up to 108-fold increase, and the lowest AD ratio is 3.5 times higher, making this difference between groups statistically significant. Similarly, in urine samples of AD and controls, there is a significant difference in the 8OHG and 8OHG/2dG (2′-deoxyguanosine) levels [42] Histone deacetylases (HDAC1) are enzymes that modulate transcription, chromatin remodeling, and DNA repair, including modulation of 8OHdG repair in the brain. HDAC1 deficiency is directly related to DNA damage accumulation and cognitive impairment in aged wild-type mice, with a similar reduced HDAC1 activity and downregulation observed in the AD mouse model (5XFAD) [43]. Pharmacological activation of HDAC1 showed benefits in 5XFAD mice and is proposed as a potential therapy for cognitive decline in aging and AD. 

Damaged DNA bases (8-hydroxyadenine, 8-hydroxyguanine, thymine glycol, Fapy-guanine, 5-hydroxyuracil, and Fapy-adenine) show increased levels in the parietal, temporal, occipital, and frontal lobe, superior temporal gyrus, and hippocampus in the AD brain, contributing to oxidative damage in neurodegeneration [44]. The oxidative damage is also observed in mitochondrial DNA in AD cases; this condition is related to progressive mitochondrial dysfunction, alteration in redox-active metal and NADPH oxidases, and overall malfunction of antioxidant systems [45]. The oxidative damage to mtDNA is also implicated in altered neurogenesis and synaptic dysfunction [46]. The extension of RNA damage was determined in AD, revealing that in the frontal cortex, around 30–70% of mRNAs are oxidized in comparison with age-matched controls [47]. Specifically, some mRNAs are highly oxidized, including Cu/Zn SOD1, carbonyl reductase 1, cytochrome *b*, presenilin 1, apolipoprotein D, transferrin, α Enolase 1, calpain small subunit 1, cadherin 18 type 2, and glutamate dehydrogenase 1. In conclusion, the oxidation of nucleic acids is extended from DNA to RNA and mtDNA in neurodegeneration; this feature implicates these alterations in the failure of critical cellular functions and the progression of AD pathogenesis.

### 3.3. Lipid Peroxidation

Lipid peroxidation is a process in which oxidants such as free radicals (ROS, RNS, H_2_O_2_, O_2_^−^) react with membrane lipids containing carbon–carbon double bonds, especially polyunsaturated fatty acids (PUFAs) (Figure 3). The reaction with H atoms from PUFAs of phospholipids generated C^•^ radicals that then reacted with O_2_ to form peroxyl free radicals (LO_2_). Consequently, a reaction with more acyl chains of phospholipids forms lipid hydroxyperoxides, and this chain reaction is repeated with additional PUFAs. Isoprostanes are a group of lipid peroxides derived from arachidonic acid (F2), eicosapentaenoic (F3), or docosahexaenoic acid (F4) acids, which are used as biomarkers of lipid peroxidation [35].

In AD brain cortex, the levels of F4-isoprostanes are elevated in comparison with age-matched controls, particularly in the occipital, temporal, and parietal lobes [48]. 4-hydroxy 2-nonenal (HNE) is one of the most abundant lipid derivatives, which is elevated in the CSF and brain in AD, but also in other neurological disorders (Parkinson’s, Huntington’s, and amyotrophic lateral sclerosis) [49]. Remarkably, the glutamate transporters are inhibited in the AD brain by oxidative damage and lipid peroxidation products such as HNE; this condition is mediated by Aβ [50]. HNE is elevated in mild cognitive impairment to the hippocampus and inferior parietal lobules, indicating that lipid peroxidation is an early event in AD pathology [51]. This trend continues to the preclinical AD stage, with abnormally elevated lipid peroxides such as HNE, malondialdehyde, and acrolein (α,β-unsaturated aldehydes) in the hippocampus, superior and middle temporal gyri, and cerebellum areas [52]. 

Lipidomics profiling (LC-MS) of plasma samples from subjects with MCI due to AD, advanced AD, and non-AD demented patients were compared to healthy participants. The analysis indicated that two compounds (15-F2t-isoprostanes and 14(RS)-14-F4-t-NeuroP) and three types of lipids (isoprostanes, isofurans, and neurofurans) have a significant difference among groups, which are produced from the oxidation of arachidonic acid, docosahexanoic, and adrenic acid [53]. Therefore, the lipidomics profiling could be exploited for AD diagnosis, especially in the early stages of the neurological disorder. Furthermore, nontargeted lipidomics of postmortem human gray matter (frontal cortex area 8) and white matter (frontal lobe centrum semi-ovale) showed that white matter profiling is characteristic of an adaptive lipid phenotype to lipid peroxidation, whereas profiling from cortex changes according to AD progression [54]. Remarkably, the main lipid classes affected were membrane structural composition, bioenergetics, antioxidant protection, and bioactive lipids.

## 4. Neuronal Death Mechanisms Related to Oxidative Stress in Alzheimer’s Disease

**Figure 4:**
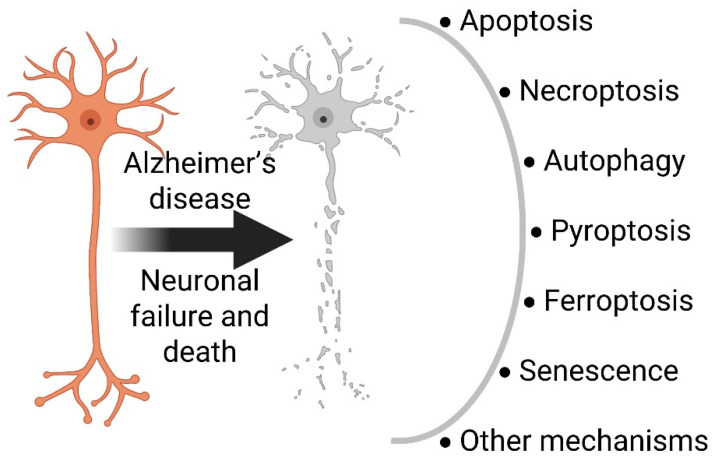
Diverse programmed mechanisms related to neuronal failure and death in Alzheimer’s disease.

Neuronal death occurs during development and pathology events; however, neurons may die through different particular mechanisms under specific circumstances [55]. Programmed cell death is critical during the development stages, and to keep homeostasis in the organisms, alterations of dysregulation of cell death mechanisms may lead to diverse diseases such as Alzheimer’s disease (Figure 4). Remarkably, neuronal loss is one of the main and most evident features of AD, but our understanding of the neuronal death mechanisms involved is still incomplete [56]. Collective information indicates occurrence of different forms of cell death in AD, including apoptosis, necroptosis, pyroptosis, autophagy, and ferroptosis in different stages of the disease. The elucidation of these complex mechanisms and the pathways involved offers alternative strategies to develop prospective therapeutics that control programmed cell death [57]. 

### 4.1. Apoptosis

Apoptosis has been extensively explored in different AD cell and animal models, and also in postmortem human brain tissues. Intrinsic apoptosis is triggered by microenvironment perturbations, including DNA damage/fragmentation, endoplasmic reticulum stress, and overload of ROS, whereas extrinsic apoptosis relates to perturbations of extracellular conditions [58]. Overall, mature postmitotic neurons seem to be resistant to apoptosis, indicating that apoptotic cell death is not the main contributor to neuronal death in AD [59,60]. 

### 4.2. Necroptosis

Necroptosis is implicated in many diseases, including neurological disorders (AD, Parkinson’s, amyotrophic lateral sclerosis, multiple sclerosis and stroke), heart failure, inflammation, and pulmonary diseases [57]. The metabolic disturbances and oxidative stress in AD promote high susceptibility of a large number of cells; this mediates neuronal loss through necrosis rather than apoptosis [60]. Necroptosis is a regulated necrosis mediated by death receptors (FAS, RIPK, MLKL, and TNFR1) or pathogen recognition receptors, which are initiated by alterations of the extracellular or intracellular microenvironment [58]. Necroptosis is activated in AD, with a positive correlation depending on the advancement of the neuropathology (Braak stage), and is inversely correlated with brain volume and cognitive scores [61]. Likewise, the biomarkers of necroptosis show an age- and region-specific increase in the brain cortex and hippocampus, which induce neuroinflammatory responses [62]. 

### 4.3. Autophagy

Autophagy-dependent cell death is a process that relies on the autophagic machinery to process or break down damaged or abnormal proteins and other cellular components. This mechanism is activated during periods of stress, starvation, in response to oxidative stress conditions, or as a major sensor of the redox signaling [63]. Autophagy is involved in Aβ generation and clearance, and is consequently linked to the accumulation of senile plaques [64] In mice lacking autophagy-related protein 7, Aβ was accumulated inside neurons with a reduced extracellular plaque formation, but with severe signs of memory impairment and neurodegeneration [65]. Autophagy is significantly impaired or dysregulated within neurons affected by AD, preceding the formation of amyloid plaques [66]. 

### 4.4. Pyroptosis

This mechanism is an inflammatory form of cell death, triggered by perturbations of extracellular or intracellular homeostasis conditions. Inflammasome multiprotein complexes are characteristic of pyroptosis; these are found in microglia and neurons during neurodegeneration, and in response to Aβ, lysosomal stress, and ROS [56]. 

### 4.5. Ferroptosis

Ferroptosis is a recently described form of programmed cell death that is characterized by iron overload, high oxidative stress, ROS overproduction, perturbations in the antioxidant systems, and lipid peroxidation [58]. In human neurons, ferroptosis pathways control neuronal responses to chronic oxidative stress, including genes related to mitochondrial and lysosome functions such as *GPX4, PSTK, SEPHS2, SEPSECS*, *mTORC1* pathway, *ASCL4, PSAP*, and *NQO1* [67] Ferroptosis results from alterations in redox homeostasis, ROS overproduction, and extensive lipid peroxidation. Likewise, neurodegeneration in AD is closely related to high oxidative stress conditions, abnormal deposition of redox-active iron, glutathione depletion, aggregation of Aβ/tau, mitochondrial dysfunction, synaptic failure, and progressive neuronal death [68,69,70].

### 4.6. Senescence

Senescence refers to a biological state by which the stressed cells stop dividing but are not dead. This complex stress response induces an aberrant cell cycle, with limited metabolic activity, and releases damage-associated molecules that may trigger inflammation and damage to neighboring cells. Senescence has been speculated to play a role in the development of AD. Transgenic AD mice display a senescence phenotype, which correlates with brain atrophy (neuronal loss) and tau deposition [71]. Senescent brain cells may contribute to the development of AD. In AD human brains, ~2% of cells showed senescence phenotype (from ~140,000 single nuclei derived from 76 cases), particularly in excitatory neurons [72]. These senescent neurons have high levels of cyclin-dependent kinase inhibitor 2D (CDKN2D/p19), as the most significant contributor to the primary senescence eigengene, also containing neurofibrillary tangles of tau. Therapeutic compounds that target senescent cells might be effective treatment options for neurodegenerative disorders. A pilot trial tested senolytics (dasatinib + quercetin) in AD patients, indicating that the therapy was well tolerated, with some mild to moderate adverse effects, but cognitive and neuroimaging endpoints did not significantly differ from controls [73].

## 5. Pharmacological Interventions to Counteract Oxidative Stress in AD

**Figure 5:**
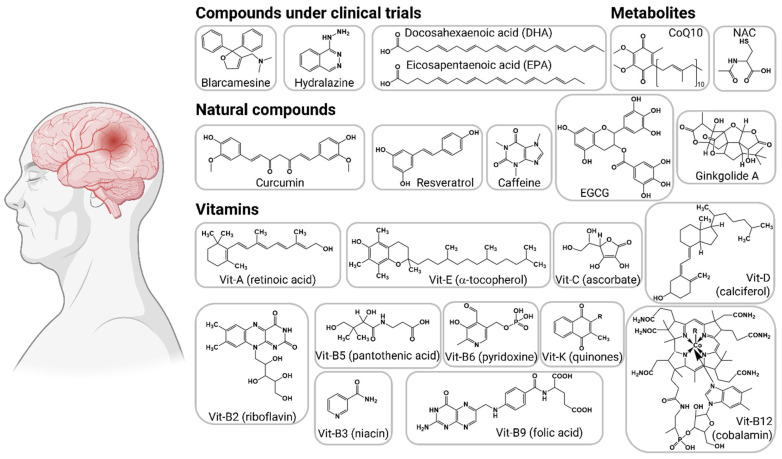
Interventions targeting oxidative stress in Alzheimer’s disease. The therapeutic compounds are organized into compounds under clinical trials, bioactive metabolites, natural compounds, and antioxidant vitamins.

The AD drug pipeline has at least 143 compounds in clinical trials, including 47 in phase 3, 82 compounds in phase 2, and 30 more in phase 1 [74]. The vast majority of compounds are focused on targeting amyloid β, synaptic plasticity, and inflammation. Only three compounds with oxidative stress as mechanism of action are in phase 3 and one is in phase 2 for AD treatment, but several other therapeutic compounds have been proposed in preclinical studies (Figure 5).

**Blarcamesine** (commercial name: ANAVEX2-73) is an agonist of the intracellular sigma-1 receptor (SIGMAR1); it inhibits mitochondrial respiratory dysfunction and prevents oxidative stress, restoring neural cell homeostasis and promoting neuroplasticity [75]. Blarcamesine treatment restored mitochondrial respiration in Aβ_25–35_-injected mice, reducing lipid peroxidation levels, and also prevented mitochondrial dysfunction and oxidative stress in hippocampus [76]. Phase 2a trial of blarcamesine (20–30 mg/day) showed safety, tolerability (clinical and pharmacokinetic), and efficacy in a 57-week study (clinicaltrials.gov: NCT02244541 and NCT02756858) [77]. This compound completed a phase 2b/3 clinical trial for the treatment of mild cognitive impairment due to AD and mild AD. In a press release, the sponsor company indicated that after 48 weeks of treatment, the compound met the primary endpoints and reduced clinical decline by 84% on the global cognitive and functional scales. Overall, treatment with blarcamesine significantly reduced cognitive decline, measured with ADAS-Cog, compared to placebo at end of treatment by 45%.

**Hydralazine**. This compound is used to treat high blood pressure. Hydralazine has neuroprotective properties against endogenous and exogenous stressors, controlling oxidative stress responses [78]. Preclinical studies showed that hydralazine reduced Aβ misfolding, oxidative lipid damage, and neurotoxicity [79,80]. This FDA-approved drug has anti-neurodegenerative properties by activating the Nrf2 pathway that includes more than 200 antioxidant proteins, rejuvenation of mitochondrial activity, and increased cellular respiratory capacity. A phase 3 clinical trial is assessing the efficacy of hydralazine in early-stage AD patients who take one of the acetylcholinesterase inhibitors (AChEI) donepezil, rivastigmine, or galantamine (clinicaltrials.gov: NCT04842552). The protocol includes a dose of hydralazine (25 mg) every eight hours for 1 year. This trial will determine the effects on cognition, functional measures, and progression of AD.

**Omega 3 (DHA+EPA)**. Supplements of omega 3 fatty acids are extracts from fish oil, enriched in docosahexaenoic acid (DHA), and eicosapentaenoic acid (EPA). These polyunsaturated fatty acids (PUFAs) exhibit neuroprotective, antioxidant, and anti-inflammatory properties [81]. Administration of omega-3 supplements has been linked to changes in mitochondria, redox conditions, and mitochondrial biogenesis [82,83]. In clinical trials, the results have been contradictory; omega-3 supplements (1.7 g DHA + 0.6 g EPA) showed no significant differences in oxidative stress in AD subjects [84]. Likewise, DHA supplements (2 g/d) showed no beneficial effects on cognitive decline in individuals with mild to moderate AD (clinicaltrials.gov: NCT00440050) [85]. Finally, omega-3 supplements (800 mg DHA/225 mg EPA) showed no perceivable effects on cognitive decline in AD (clinicaltrials.gov: NCT00672685) [86]. Furthermore, a purified form of an omega fatty acid, ethyl eicosapentaenoic (IPE), has demonstrated some effects in mitochondrial lipids and reduction of oxidative damage [87], but in clinical settings, these effects were not significant in AD patients [88]. A phase 2/3 clinical trial of IPE is ongoing to evaluate its efficacy on cognitive performance and biomarkers in subjects with AD (clinicaltrials.gov: NCT02719327).

**Natural herbal products.** Several natural herbal supplements have attracted interest for their potential neuroprotective properties and targeting different pathological mechanisms associated with AD [89]. Usually, these natural products are a mixture or extract containing multiple bioactive compounds that synergistically may exert multiple neuroprotective, antioxidant, anti-inflammatory, anti-Aβ, or nootropic mechanisms. Some products have achieved preclinical and clinical evaluation to confirm their effectiveness in preventing and attenuating neurodegeneration or symptomatology of dementia.

**Curcumin.** This polyphenol found in turmeric spice has long been used for medical purposes. The antioxidant properties of curcumin include the removal of reactive oxygen and nitrogen species; metal chelation; regulation of antioxidant enzymes; anti-inflammatory; reduction of malondialdehyde; and overall increase in the total antioxidant potential [90]. Curcumin binds to Aβ, inhibiting its aggregation into fibrils and plaques, as observed in in vitro and in vivo Tg2576 mice AD models [91]. A phase 2 clinical trial examined the safety and tolerability of a curcumin formulation (Curcumin C3 Complex) on patients with mild to moderate AD. In the 24-week trial, the patients received 2–4 g/d or placebo to determine effects in cognition test (ADAS-Cog and MMSE), executive function (ADCS-ADL), and AD biomarkers (plasma Aβ, tau, and isoprostanes). The trial concluded that curcumin was generally well tolerated, although some participants showed gastrointestinal symptoms. Overall, the trial showed no clinical or biological efficacy in the outcome measures [92].

**Resveratrol**. This bioactive compound found in grapes and red wine has been extensively studied for its neuroprotective, anti-inflammatory, immunomodulatory effects, and antioxidant properties. Resveratrol is a potent activator of sirtuins (SIRT1), deacetylases that regulate energy (NAD/NADH), and glutamate receptors [93,94]. The neuroprotective mechanisms of resveratrol are related to inhibition of Aβ aggregation and inhibition of free radicals [95,96]. Clinical trials of resveratrol indicated that this polyphenol is well tolerated by young and elderly subjects, when administered 200 mg at 8 h intervals, but it is rapidly metabolized and excreted [96]. In the healthy elderly, resveratrol increases blood flow in a dose-dependent manner but without changes in cognitive function [97]. The phase 3 clinical trial tested a dietary supplement of resveratrol (5 mg) with glucose (5 g) and malate (5 g), administered twice a day for 1 year to subjects with mild to moderate AD (clinicaltrials.gov: NCT00678431). The supplement showed less deterioration in cognitive measures, but none of the cognitive scores reached statistical significance [98].

Likewise, grape seed extract containing polyphenols has been proposed as dietary supplement with potential used in neurodegeneration. In animal models, this grape seed extract has effects on Aβ and tau phenotypes [99]. The ongoing phase 2 clinical trial will establish the safety and pharmacokinetic of grape seed extract in AD subjects (clinicaltrials.gov: NCT02033941).

**Caffeine**. Caffeine, along with its catabolic products theobromine and xanthine, is recognized as an antioxidant, but also with prooxidant activities [100]. The antioxidant activity relates to scavenging of free radicals and inhibition of hydroxyl radicals, whereas prooxidant activity occurs by reactivity with nucleic acids and with transition metal ions. Neuroprotective properties of caffeine/coffee are related to antagonistic activity against adenosine receptors in the central nervous system. A clinical trial examined the neuroprotective effects of caffeine (over three cups per day for 4 years) in elderly subjects. Caffeine consumption was associated with less decline in verbal and visuospatial memory in elderly women, but had no perceivable effects on cognitive decline in men [101]. Also, the caffeine consumption did not reduce dementia risk, but will be further explored to prolong or maintain the period of mild cognitive impairment in elderly subjects. A phase 3 trial is currently evaluating the effects of caffeine supplement (100–400 mg capsules) administered for 30 weeks to treat symptoms of AD (clinicaltrials.gov: NCT04570085).

**Epigallocatechin gallate** (EGCG) is a flavonoid (polyphenolic) compound present in green/black tea and known for its antioxidant properties. Oral administration of EGCG (50 mg/kg) for 4 months to APP/PS1 mice significantly attenuated cognitive deficits and reduced neuroinflammation and Aβ in the hippocampus [102]. In APP/PS1 mice under a high-fat diet, the administration of EGCG improved insulin signaling and memory deficits, correlating with a reduction in Aβ and inflammatory responses [103]. A functional drink enriched with polyphenols (green tea, vitamins, apple, and lemon) was evaluated in patients with moderate AD. After 8 months of treatment, the antioxidant drink reduced the levels of plasma total homocysteine, but had no effects on cognitive measures [104]. Several trials for EGCG have produced inconsistent results. EGCG supplementation was shown to be overall safe and tolerated (with sporadic liver damage), but without significant effects on the primary outcomes [105]. An EGCG supplement is under clinical trial phase 2/3 to determine its effectiveness in AD patients (clinicaltrials.gov: NCT00951834). Subjects will receive up to 800 mg EGCG add-on to donepezil for up to 18 months to determine safety, tolerability, and effectiveness on cognitive tests and brain atrophy.

**Ginkgo biloba**. This plant has been used for traditional medicine for centuries. The extract of *Ginkgo biloba* contains a mixture of bioactive components like trilactonicditerpenes, ginkgolides, and several flavonoids, such as quercetin, kaempferol, isorhammetins, trilactonicsesquiterpene, and proanthocyanidins [106]. The standardized extract of *Ginkgo biloba*, known as EGB761, has shown antioxidant properties, including free radical scavenging, reduction of ROS, metal chelation, and upregulation of antioxidant enzymes (superoxide dismutase, glutathione reductase, and gamma-glutamylcyateinyl synthase) [106,107]. *Gingko biloba* EGB761 has been tested in at least 36 trials for dementia or cognitive decline [108]. Even though there have been inconsistent results, due to variable time of treatments, population, or dosage, the herbal supplement is generally safe, well tolerated, and in some of these trials is reported to have benefits for cognition, mood, depression, and executive function. A randomized controlled trial of EGB761 for prevention of dementia was performed with more than 3000 elderly participants. Over a 5-year period, the participants received 120 mg of EGB761 twice per day, indicating no significant differences in cognitive decline (memory, attention, visuospatial abilities, language, and executive functions) between treated and placebo groups [109,110,111].

**Vitamin supplements**. The brain is vulnerable to oxidative stress, leading to neuronal damage and functional deficiencies. Antioxidant compounds are proposed for neuroprotection of the brain from oxidative insults [112]. Dietary vitamin supplements are promoted for cognitive protection, but with no clear scientific evidence. Serum antioxidant vitamins and carotenoids may protect against neurodegeneration. Data analysis of Medicare and Medicaid indicated that serum vitamins (A, C, and E) and carotenoids (α-carotene, β-carotene, lutein+zeaxantine, β-cryptoxanthin, and lycopene) were associated with reduced risk of all-cause dementia, but further studies are required to fully determine time-dependent neuroprotection effects of supplements [113]. A randomized controlled 3-year trial showed that a multivitamin-mineral supplement (Centrum Silver, Pfizer) has potential efficacy to improve cognition, memory, and executive function in older adults (clinicaltrials.gov: NCT04582617) [114,115]. Additional studies will follow to confirm these findings in diverse cohorts and to identify mechanisms for multivitamin-mineral effects.

**Vitamin A**. The bioactive form of vitamin A, all-*trans*-retinoic acid, is recognized as a transcriptional regulator. Vitamin A is an indirect antioxidant, regulating the transcription of the canonical antioxidant responses [116]. All-*trans*-retinoic acid is capable to induce synaptic plasticity in the adult brain cortex by promoting coordinated and functional changes of the excitatory synapses of superficial pyramidal neurons [117]. Deficiency in vitamin A affects cognitive functions, and promotes Aβ peptide production and neuritic plaque formation, and also significantly exacerbates memory deficits in transgenic AD mice [118]. Retinoic acid helped to reduce Aβ deposition, decreased activation of microglia and astrocytes, and improved spatial learning and memory in transgenic APP/PS1 mice after an intraperitoneal 8-week treatment (20 mg/kg, three times weekly) [119]. For these reasons, vitamin A has been proposed as a potential treatment for neuropsychiatric disorders such as AD. There are no clinical trials reported for vitamin A treatments for AD and related dementia, but a pilot trial with bexarotene, a class of retinoid medication, was performed with 20 patients with AD. The administration of 300 mg of bexarotene for 4 weeks showed that only ApoE4 noncarriers had a significant reduction in brain amyloid, whereas in ApoE4 carriers there were no significant changes (clinicaltrials.gov: NCT0178272) [120].

**Vitamin E**. This is a group of lipophilic compounds, including α-, β-, γ-, and δ-tocopherols and α-, β-, γ-, and δ-tocotrienols. Vitamin E is recognized as a lipophilic antioxidant that protects polyunsaturated fatty acids in cell membranes and acts as a transcriptional regulator [116]. Studies in AD animal models suggest that vitamin E supplementation decreases oxidative stress and may improve cognitive and memory decline [121]. Also, when this vitamin is combined with other antioxidant compounds, the antioxidant and anti-inflammatory effects are increased. However, in some instances the metabolic benefits are not significant, indicating that the clinical benefit of vitamin E supplement is under debate. The neurotoxic and oxidative stress effects (ROS production) of Aβ_42_ were prevented with vitamin E addition to primary rat embryonic hippocampal cells [122]. Higher brain α- and γ-tocopherol levels are associated with lower AD prevalence; this is related to lower total and activated microglia density in cortical brain regions [123]. The effect of vitamin E and memantine on functional decline in AD was determined in a phase 3 randomized trial. Patients with mild to moderate AD received 2000 IU/d of α-tocopherol, 20 mg/d memantine, their combination, or a placebo for up to 48 months (clinicaltrials.gov: NCT00235716). The group of α-tocopherol showed slower functional decline in comparison with placebo; also, there were no significant differences in the memantine group or memantine/α-tocopherol group [124]. These findings suggest that α-tocopherol reduced cognitive decline in subjects with mild to moderate AD.

**Vitamin C**. Ascorbic acid (vitamin C) functions as an antioxidant, protecting biomembranes from free radical damage [125]. The antioxidant reaction of vitamin C involves the donation of a single reducing equivalent (oxidation), forming monodehydroascorbate which reacts preferentially with radicals instead of with nonradical compounds [126]. Vitamin C has neuroprotective properties, including preclinical and clinical studies of hypoxic brain injury, intracerebral hemorrhage, ischemic stroke, and neurodegeneration [127]. In these studies, the median maximum daily dose of exogenous vitamin C was 750 (500–1000) mg or 10 mg/kg for adults. In 5XFAD mice, a high dose of vitamin C supplementation to the drinking water (3.3 g/L) reduced amyloid plaque deposition (cortex and hippocampus), reduced BBB disruption and mitochondrial alteration, and also reduced pathological changes [128]. The antiaging or neuroprotective effects of vitamin C are also related to reduction of inflammatory responses, chelation of metal ions (iron, copper, and zinc), and reduction of Aβ fibrillogesis [129]. There are at least 12 studies quantifying the plasma levels of vitamin C in relation to AD pathophysiology [130]. Overall, the deficiency of vitamin C is involved in AD progression; maintaining healthy vitamin C levels may have a protection against cognitive decline, whereas the supplementation is still under scrutiny to fully determine plausible preventive or therapeutic benefits. A pilot phase 1 trial evaluated the safety and effectiveness of antioxidant regimens in patients with mild to moderate AD (clinicaltrials.gov: NCT00117403). The administration of antioxidants (800 IU/d of vitamin E + 500 mg/d vitamin C + 900 mg/d α-lipoic acid) for 16 weeks did not change AD biomarkers (CSF Aβ/tau), but it showed a reduction in F2-isoprostane levels related to oxidative stress in the brain [131]. 

**Vitamin D**. Vitamin D, also referred to as calciferol, is a fat-soluble vitamin present in fish and liver, and is produced endogenously in the skin upon interaction with ultraviolet sunlight. In foods and dietary supplements, vitamin D is present in two main forms, D_2_ (ergocalciferol) and D_3_ (cholecalciferol), differing only in their side-chain structures. The antioxidant properties of vitamin D are related to free radical and ROS scavenging, reduction of lipid peroxidation, and reduction of oxidative stress biomarkers [132,133]. Evidence indicates that vitamin D has a neuroprotective role, reducing neurological injury and neurotoxicity, also crucial role in neurodevelopment, proliferation, differentiation, neurotropism, neuroprotection, neurotransmission, and neuroplasticity [134,135]. A clinical trial with 210 AD patients studied the effect of a 12-month vitamin D supplementation (800 IU/d) on cognitive function and AD biomarkers [136]. The trial concluded that vitamin D supplementation improved cognitive function and decreased AD-related biomarkers (plasma Aβ, APP, BACE1, *APP* mRNA, and *BACE1* mRNA). Another recent trial indicated that vitamin D supplementation was associated with significantly longer dementia-free survival and 40% lower dementia incidence rate than no treatment [137]. Interestingly, the vitamin D effects were significantly greater in ApoE4 noncarriers versus carriers. In subjects with genomic vitamin D receptor (VDR)/RXR complex variant, these effects of vitamin D supplementation were negative, causing a worsening in AD progression, including Aβ deposition and exacerbated symptomatology [138].

**Vitamin K**. This is a generic name for a group of lipophilic naphthoquinone compounds that include phylloquinone (vitamin K1) and menaquinones (vitamin K2 and K3) with unsaturated isoprenyl side chains which are designated as MK4 through MK-13, based on the length of their side chain. These compounds are found in green leafy vegetables, animal products, in bacteria, and in fermented foods. The antioxidant properties of vitamin K are related to protection of cell membranes from free radicals. Vitamin K protects neurons and oligodendrocytes from oxidative stress, excitotoxicity, inflammation, and neuronal cell death in animal models [139]. In elderly patients, higher vitamin K intakes are associated with better cognitive function, lower odds of dementia or mild cognitive impairment (17–20%), lower AD global pathology scores (14–16% lower Braak stage), and fewer tau neuronal tangles deposition [140]. 

**Vitamin B complex**. This group of compounds has diverse functions in the body, from enzymes cofactors to energy production, mitochondrial activity, amino acid metabolism, and transporting oxygen and energy. There are eight B vitamins, commonly known as B complex, including B1 (thiamin), B2 (riboflavin), B3 (niacin), B5 (pantothenic acid), B6 (pyridoxine), B7 (biotin), B9 (folic acid), and B12 (cobalamin). Some of the members of this group of vitamins have antioxidant potential and may reduce oxidative stress. A meta-analysis (total of 21 clinical trials) indicated that vitamin B supplements might delay or slow cognitive decline of elderly adults, but cannot improve the information processing speed, episodic memory, or executive function in patients with MCI or elderly without cognitive impairment [141].

**Vitamin B2** (riboflavin) is involved in glutathione metabolism, which is the main antioxidant compound in cells. Riboflavin supplementation (5 mg/d) activates the enzyme glutathione reductase [142], and is a neuroprotective compound, reducing oxidative stress, mitochondrial dysfunction, neuroinflammation, and glutamate excitotoxicity [143]. Dietary intake of riboflavin showed protective effects for global cognitive function and verbal memory domain in middle-aged and elderly participants (4-year trial) [144]. **Vitamin B3** (niacin) regulates lipid metabolism, including lipoproteins, cholesterol metabolism, inflammatory responses, and oxidative stress [145,146]. Niacin is required for oxidative reactions, energy production, and other nonredox signaling pathways, including neuroprotective effects [147]. Dietary intake of niacin reduces the risk of AD and protects against cognitive decline, as observed in 5xFAD mice (dose 100 mg/kg for 30 d) [148]. In elderly subjects, higher food intake of niacin was associated with a slower annual rate of cognitive decline and AD, as observed in a 9-year study (3-year study and 6-year follow up) [149]. **Vitamin B5** (pantothenic acid) is involved in lipid metabolism, coenzyme Q biosynthesis, and ATP production, and also protects cells against oxidative stress by regulating glutathione metabolism and cellular repair. The deficiency of vitamin B5 affects brain regions known to be severely damaged in AD cases (hippocampus, entorhinal cortex, and middle temporal gyrus) in comparison with aged-matched controls, indicating that its deficiency may cause neurodegeneration and dementia [150]. **Vitamin B6** (pyridoxine) is implicated in the metabolism of amino acids, glycogen, and lipids, but the exact antioxidant mechanisms of vitamin B6 have not been elucidated. Pyridoxine reacts with peroxide radicals serving as scavengers and limiting lipid peroxidation. Also, pyridoxine plays an indirect antioxidant role as coenzyme of the glutathione antioxidant system [151]. Pyridoxine and its derivatives have antihypertensive and neuroprotective properties, protecting neurons against ischemia and glutamate-induced neurotoxicity [152]. Vitamin B6 deficiency is linked to accumulation of homocysteine, which is associated with an increased risk of AD. **Vitamin B9** (folic acid) has antioxidant activity, increasing cellular total antioxidant capacity and reducing ROS formation [153]. The role of folic acid in prenatal development has been extensively investigated, having critical roles in neurodevelopment and neuroprotective effects [154]. Serum folate deficiency was associated with higher risks of dementia and all-cause mortality in the elderly (60–75 years) [155], but vitamin B9 deficiency is correlated with other metal symptoms, especially depression and cognitive decline in epileptic, neurological, psychiatric, geriatric, and psychogeriatric populations [156]. Folic acid supplementation (400 μg) significantly improved cognitive function and reduced inflammation responses in elderly subjects with MCI after 12 months of intervention [157]. Patients with AD that received folic acid supplements (1.25 mg/d) and donepezil showed lower levels of Aβ and inflammatory biomarkers after a 6-month intervention [158]. **Vitamin B12** (cobalamin) possesses antioxidant properties by direct scavenging of ROS, superoxides, regulation of glutathione levels, modulation of cytokines, and inflammation responses [159]. Cobalamin and other B-vitamins have neurotropic and regenerative properties, promoting nerve cell survival and remyelination [160]. Low levels of B12 are linked to impaired cognition and memory and also other nerve symptoms such as tingling, numbness, and poor myelination. Supplementation of B12 was shown to improve symptoms and cognitive outcomes in patients with MCI [161]. In elderly with confirmed AD, supplementation with B12 showed improvement in cognition, memory, and symptoms in a subgroup, whereas in others, it showed no benefit [162]. This difference in response could be related to the stage of neuropathology at the moment of the intervention. A phase 2/3 trial in patients with MCI showed that subjects with low-normal vit-B12 have significantly poorer learning ability and cognitive performance in comparison with patients with high-normal vit-B12. Furthermore, microstructure integrity of the hippocampus was lower in the low vit-B12 group, correlated with the effects in cognition and memory (clinicaltrials.gov: NCT01219244) [163].

We can conclude that optimal levels of vitamin B complex are needed to maintain neuronal metabolism and reduce cognitive decline. The clinical benefits of supplementation in AD pathology still are unclear and under investigation, but evidence suggests that these vitamins B (B1, B6, and B12) serve in nerve regeneration and myelin formation [160]. Furthermore, a pilot clinical trial showed that B-vitamins supplementation (0.8 mg folic acid, 20 mg vitamin B6, and 0.5 mg vitamin B12) slowed shrinkage of the whole brain (atrophy) over 2 years, particularly in the brain regions related to cognition and AD (hippocampus, cortex) [164]. 

**Coenzyme Q10** (CoQ10 or ubiquinone) is one of the most significant lipid antioxidants, preventing or neutralizing the generation of free radicals, ROS, and potential oxidative damage to cellular components, particularly in the mitochondria [165]. CoQ10 has demonstrated neuroprotective properties, serving as a potent free radical scavenger in lipid and mitochondrial components. Administration of CoQ10 to rats (12–24 months) resulted in a significant increase in intracerebral CoQ concentrations, attenuating striatal lesions and increasing lifespan [166]. In a PD rat model, the intrastriatal administration of CoQ10 (25–40 μg/mL) prevented dopaminergic neuron degeneration, demonstrating neuroprotective effects [167]. In AD mice (Tg19959), the CoQ10 treatment resulted in decreased levels of protein carbonyls, decreased Aβ deposition, and improved cognitive performance [168]. The therapeutic potential of CoQ10 has been evaluated in eight studies in patients with AD [169]. The findings are contradictory: some trials showed improvement and others did not; this has prevented the advancement of additional clinical trials. For example, the phase 1 trial (clinicaltrials.gov: NCT00117403) evaluated the dose of 400 mg of CoQ10, and compared with other antioxidants (800 IU vit E + 500 mg vit C + 900 mg α-lipoic acid) in 75 patients with mild to moderate AD [131]. Overall, the 16-week treatments of antioxidants did not change AD biomarkers (CSF), and in some cases the treatment caused a faster cognitive decline.

**N-acetyl cysteine** (NAC) is a cysteine prodrug which is used as pharmacological antioxidant and cytoprotectant. It is generally considered a poor scavenger of oxidants and free radicals, but NAC can inactivate ROS, H_2_O_2_, O_2_^−^, and lipid peroxides through reaction with thiolate residues [170,171]. Additionally, NAC is a precursor of glutathione, providing Cys residues through NAC deacetylation. Neuroprotective properties of NAC were demonstrated in primary rat hippocampus neurons dosed with H_2_O_2_; the mechanism of action was related to inhibition of ROS, downregulation of MAPK signal transduction, and tau phosphorylation [172]. A pilot trial with patients with probable AD tested the efficacy of NAC, observing significant differences only for a subset of cognitive tasks [173]. A phase 2 clinical trial tested a nutritional formulation with NAC for 3–6 months in individuals diagnosed with AD (clinicaltrials.gov: NCT01320527). Overall, the trial indicated that NAC maintained or slightly improved cognitive performance and mood/ behavior of the AD patients [174]. Another phase 2 trial tested the effects of a dietary supplement with cofactors NAC, L-carnitine tartrate, nicotinamide riboside, and serine in AD patients (clinical trials.gov: NCT04044131). After treatment (1-month daily dose of 12.35 g containing 2.55 g NAC, 3.73 g L-carnitine, 1 g nicotinamide riboside, and 12.45 g serine, then two doses from day 28 to day 84), the patients showed enhanced cognitive functions, improved clinical parameters (hippocampal volumes and cortical thickness based on imaging), and increased plasma levels of NAD and glutathione [175].

## 6. Conclusions

Cumulative evidence confirms the central role of oxidative stress responses in the onset and progression of Alzheimer’s disease and related dementia. Nevertheless, oxidative stress is neglected as an integral part of the histopathological features of AD, and is generally recognized as a consequence of the main cellular and molecular mechanisms of this neurological disorder. As discussed here, oxidative stress has a critical role in the activity of Aβ, progressive damage of cellular components, and the activation of programmed cell death mechanisms. A few therapeutic compounds are currently under clinical trials to overcome oxidative stress in AD and related dementia, and several alternative active compounds or supplements are under preclinical and clinical evaluation to determine their effectiveness. As a multifactorial disease, it is expected that multitarget therapies will be designed for AD, including oxidative stress as a critical component that complements the main therapies against Aβ, tau, neurotransmitters, and neuroinflammation.

## Data Availability

Not applicable.
